# A Rare Presentation of Coinfection: Dengue Virus and Hepatitis A Virus

**DOI:** 10.1155/2019/9782892

**Published:** 2019-08-18

**Authors:** I. Ranathunga, L. S. Kannangara, K. A. S Bandara, S. B. Gunatilake

**Affiliations:** ^1^Registrar in Medicine, Professorial Medical Unit, Colombo South Teaching Hospital, Dehiwala-Mount Lavinia, Sri Lanka; ^2^Senior Registrar in Medicine, Professorial Medical Unit, Colombo South Teaching Hospital, Dehiwala-Mount Lavinia, Sri Lanka; ^3^Professor of Medicine, Department of Medicine, Faculty of Medical Sciences, University of Sri Jayewardenepura, Nugegoda, Sri Lanka

## Abstract

Dengue fever caused by dengue virus is a common tropical infection transmitted by the mosquitos *Aedes aegypti* and *Aedes albopictus*. Four strains of the genus flavivirus is responsible for the epidemics of varying severity. Hepatitis A caused by hepatitis A virus is spread by faecal-oral route. The culprit virus is a hepatovirus. Coinfection with dengue virus and hepatitis A virus is rare and is a diagnostic as well as management challenge to the medical professional. We report a patient who presented to us with dengue virus and hepatitis A virus coinfection.

## 1. Introduction

Dengue fever (DF) is a common arthropod-borne tropical infection. It is caused by four serotypes of the genus flavivirus [1]. The incidence is high with up to 50 million infections occurring annually with only 5,00,000 developing dengue haemorrhagic fever (DHF) [1]. Hepatitis A caused by hepatitis A virus is transmitted by faecal-oral route, and annually 1.4 million new cases occur [1]. Coinfection with dengue virus and hepatitis A virus is rare entity and is a diagnostic as well as a management challenge to the medical professional. Coinfection with other infections such as leptospira, malaria, hepatitis E, and typhoid fever has also been documented [2].

## 2. Case Report

A 34-year-old previously healthy male presented with high grade fever associated with constitutional symptoms of five days' duration. He was complaining of right hypochondrium pain and tea-coloured urine for two days associated with yellowish discolouration of the eyes. He had severe loss of appetite to food and water, but his bowel habits had been normal throughout the illness. On admission, he was afebrile with normal vital signs but was deeply icteric. His abdominal examination revealed a tender, mild hepatomegaly while his cardiovascular, respiratory, and nervous system examination was normal.

His full blood count revealed a white cell count of 3 × 10^3^ *μ*L with a platelet count of 116 × 10^3^ *μ*L. His aspartate aminotransferase (AST) and alanine aminotransferase (ALT) were 5162 U/L and 3964 U/L, respectively. Initial total bilirubin was found to be 61.7 *μ*mol/L with an increased direct fraction. His prothrombin time/international normalized ratio (PT/INR) and serum protein were normal throughout the illness. His dengue IgM antibody done by chromatographic immunoassay was positive on the 6^th^ day of the illness. Hepatitis A IgM antibody done by the ELISA technique was positive on day 6 of the illness, while hepatitis A IgG antibody was negative. Other investigations done during the hospital stay are summarized in Table 1.

During the course of the illness, the patient was closely monitored for features of development of liver failure while continuing with the precritical monitoring of the dengue fever. The patient's hospital stay was uneventful, and he did not develop features of acute liver failure or features of plasma leakage as in DHF. He was discharged on day 10 of the illness. On follow-up, his AST, ALT, and bilirubin have become normal.

## 3. Discussion

DF is caused by dengue virus and transmitted by vector *Aedes* mosquito. Hepatitis A infection is caused by hepatitis A virus and transmitted via faecal-oral route. Although both infections are common in the population occurring as isolated infections, coinfection is rare.

Coinfection of dengue virus with other infections has been documented in the past [2]. DF per se is associated with hepatic involvement which ranges from minor alterations in the aminotransferase levels to acute hepatitis [3]. DHF is associated with a greater incidence of hepatitis and fulminant hepatitis than simple dengue fever [3]. The pathogenesis of liver involvement in DF is still poorly understood. Direct viral invasion of the liver cells or products of host immune response acting on liver cells is thought to contribute to the liver cell injury [3]. The elevation of transaminases is mild to moderate in most cases of DF, and the level of AST is greater than that of ALT [3]. The levels decrease to normal levels usually within six weeks of resolution of infection [4]. However, jaundice is an uncommon finding in DF [4].

Liver biopsies performed on hepatitis A patients have revealed hepatocellular necrosis with ballooning, eosinophilic degeneration, and infiltration of mononuclear cells, accounting for the liver injury due to direct viral invasion and cellular immune response [5]. Serum AST/ALT levels both rise rapidly during the prodromal period, reach peak levels, and then decrease by approximately 75% per week. Serum bilirubin concentrations reach peak levels later and decline less rapidly than serum aminotransferases. Complete clinical recovery with restoration of normal serum bilirubin and aminotransferase values is usually achieved by 6 months [6].

Differentiating between the two infections and determining the possibility of coinfection are important in the management of an acutely ill patient with hepatitis. A patient presenting with haemoconcentration, thrombocytopenia, and plasma leakage in the presence of features of hepatitis should alert the clinician about the possibility of DF, while on the contrary, elevated bilirubin levels and deranged coagulation profile should lead towards the possibility of viral hepatitis as they are usually unchanged in DF [4].

## Figures and Tables

**Table 1:** Investigations done during the hospital stay.

Date	14/01/16	15/01/16	16/01/16	17/01/16	18/01/16
White cell count (×10^3^)	3	3.77	4.86	4.41	4.13
Haematocrit (%)	48	48.6	45.6	43.1	41.5
Platelet count (×10^3^)	116	108	141	152	131
AST (U/L)	5162			2686	635
ALT (U/L)	3964			2510	250
Total bilirubin (*μ*mol/L)	61.7	58.5	55.5	71.4	25
Direct bilirubin (*μ*mol/L)	49.1	45.8	48.8	60.7	18
